# Supplementary material for: A Critical View of the Application of the APEX Software (Aqueous Photochemistry of Environmentally-Occurring Xenobiotics) to Predict Photoreaction Kinetics in Surface Freshwaters
Source: Molecules. 2019 Dec 18;25(1):9. doi: 10.3390/molecules25010009 (PMC7017383; doi:10.3390/molecules25010009)
Supplement: Supplementary file 1 [file molecules-25-00009-s001.zip › 3.2.4_gcc-4.4.0.zip!/3.2.4_gcc-4.4.0/3.2.4_gcc-4.4.0/doc/octave/html/interpreter/Cell-Arrays-in-Oct_002dFiles.html]

Cell Arrays in Oct-Files - Untitled


Next: Structures in Oct-Files,
Previous: Character Strings in Oct-Files,
Up: Oct-Files


---

#### A.1.4 Cell Arrays in Oct-Files

Octave's cell type is equally accessible within oct-files. A cell
array is just an array of `octave_value`s, and so each element of the cell
array can then be treated just like any other `octave_value`. A simple
example is

```
```
     #include <octave/oct.h>
     #include <octave/Cell.h>
     
     DEFUN_DLD (celldemo, args, , "Cell Demo") 
     {
       octave_value_list retval;
       int nargin = args.length ();
     
       if (nargin != 1)
         print_usage ();
       else
         {
           Cell c = args (0).cell_value ();
           if (! error_state)
             for (octave_idx_type i = 0; i < c.nelem (); i++)
               retval(i) = c.elem (i);
         }
     
       return retval;
     }
```
```

Note that cell arrays are used less often in standard oct-files and so
the Cell.h header file must be explicitly included. The rest of this
example extracts the `octave_value`s one by one from the cell array and
returns be as individual return arguments. For example consider

```
     [b1, b2, b3] = celldemo ({1, [1, 2], "test"})
     =>
     b1 =  1
     b2 =
     
        1   2
     
     b3 = test
```
